# The Brazilian private pharmaceutical market after the first ten years of the generics law

**DOI:** 10.1186/s40545-019-0179-9

**Published:** 2019-08-14

**Authors:** Andréa Dâmaso Bertoldi, Anita K. Wagner, Isabel Cristina Martins Emmerick, Luisa Arueira Chaves, Peter Stephens, Dennis Ross-Degnan

**Affiliations:** 1 Postgraduate Program in Epidemiology, Federal University of Pelotas, Rua Marechal Deodoro, 1160 3º piso, Pelotas, RS CEP 96.020-220 Brazil; 2 Department of Population Medicine, Harvard Medical School and Harvard Pilgrim Health Care Institute, 401 Park Drive, Suite 401, Boston, MA 02215 USA; 3 Department of Surgery, Division of Thoracic Surgery, University of Massachusetts Medical School, 67 Belmont street, #201, Worcester, MA 01605 USA; 4 Pharmacy School, Federal University of Rio de Janeiro, Campus Macaé, Av. Aluizio da Silva Gomes, 50, Novo Cavaleiros, Macaé, RJ 27930-560 Brazil; 5 IQVIA, 210 Pentonville Rd, London, N1 9JY UK

**Keywords:** Generic medicines; pharmaceutical policy, Market share, Prices, Brazil

## Abstract

**Objectives:**

To describe changes in the private market for selected originators, branded generics (‘*similares*’), and generic products during the 10 years following passage of the Brazilian Generics Law.

**Methods:**

We analyzed longitudinal data collected by IQVIA® on quarterly sales by wholesalers to retail pharmacies in Brazil from 1998 through 2010, grouped by originators, branded generics, and generic products in three therapeutic classes (antibiotics, antidiabetics, and antihypertensives). Outcomes included market share (proportion of the total private market volume), sales volume per capita, prices and number of manufacturers by group.

**Results:**

In the private market share, generics became dominant in each therapeutic class but the speed of uptake varied. Originators consistently lost most market share while branded generics varied over time. By the end of the study period, generics were the most sold product type in all classes, followed by branded generics. The number of generic manufacturers increased in all classes, while branded generics increased just after the policy but then decreased slowly through the end of 2010. For approximately 50% of the antibiotics analyzed, branded generics and generics had lower prices than originators. For antidiabetics, branded generic and generic prices were quite similar during the period analyzed. Price trends for the various subclasses of antihypertensive exhibited very different patterns over time.

**Conclusion:**

Sales of branded generics and originators decreased substantially in the three therapeutic classes analysed following the introduction of the generics policy in Brazil, but the time to market dominance of generics varied by class.

## Introduction

The World Health Organization considers equitable access to safe and affordable medicines as vital to the attainment of the highest possible standard of health by all [1, 2]. Strategies aimed at improving access to essential medicines have been adopted in many countries. Of special relevance are strategies that aim to guarantee not only the availability of medicines [1]. Despite those initiatives, access to medicines remains an important issue to be addressed in low and middle-income countries, where out-of-pocket expenditures for medicines are an important economic burden [3].

In Brazil, access to health care and medicines is recognized as a citizen’s constitutional right and the government’s responsibility [4]. The Unified Health System (SUS – ‘*Sistema Único de Saúde’*) provides free of charge all medicines, which are part of the national list of essential medicines (RENAME – ‘*Relação Nacional de Medicamentos Essenciais*’). However, low availability in the public sector leads to high out-of-pocket expenditures for medicines in the private sector [5]. Data from the household expenditures survey shows that, with 47% of total household expenditures, health care accounts for the fourth most important group of expenses, with a greater burden among the poorest [6]. Although the majority of the Brazilian population uses SUS, around 27% of all households pay for private health insurance [7], which in Brazil usually does not cover outpatient medicines [8]. Increasing the affordability of medicines is a key goal of Brazilian health policy [3, 8]. Ensuring access to medicines is especially relevant among the poorest population. The catastrophic health expenditure in Brazil between 2002–2003 and 2008–2009 increased significantly, becoming 5.20 times higher among the poorest [9].

The Brazilian pharmaceutical market is one of the worldwide leaders in terms of total expenditures [10, 11], which is a consequence of the size of the country, characteristics of the market, and pharmaceutical policies. Some regulatory action by the Brazilian government guide the first 10 years of implantation of the generics Law. First, the generics Law and second, the regulation of controlling price cap, enforced after 1999, together with the creation of the new Medicines Regulatory Board (CMED) after 2003. In addition, the regulation of “*similar*” medicines in 2007, also had effects on the market [12].

With regard to price control, CMED implemented an annual price readjustment procedure, with a base date in March, according to a price-cap regulation model. CMED also controls the entry prices of new medicines in the Brazilian market according to specific rules by type of medicine. The manufactory price of an incoming generic drug should correspond to 65% of the price of the reference drug at its market launch. The Resolution 2 (2004) of the CMED provides that, at the time of registration of a generic product, its price must be at least 35% lower than the price of the pioneer branded drug to which it refers. These regulations, impacts on the market dynamic and differ from other countries [12].

The Generic Medicines Law in Brazil was approved in February, 1999 (Law 9787) [13], three years after Brazil began to respect the patent rights of originator medicines [14]. One year later (February, 2000), the first generic medicines were approved. Since then, there have been three types of medicines in the Brazilian Market. Originators are the innovator products; generics are products considered interchangeable (bioavailability and bioequivalence must be proven) with the originator and are commercialized with no brand name; and ‘*similares*’ are products marketed under a trade name, comparable to branded generics [15] described in the international literature. However, since 2003, all ‘*similar*’ medicines were required to prove their bioequivalence in order to renew their registration [16].

Since the 1990’s, many regulations aimed at assuring the quality of the medicines available in the market have been implemented (Table 1). Encouraging market entry of generics was one strategy to increase access to high-quality and low-cost medicines [17]. Another one was the advent of the Popular Pharmacy Program, which allows some private pharmacies to sell medicines at very low costs, with the largest share of the cost paid by the government. In 2011, the program also started the free provision of medicines for treating hypertension and diabetes [8].

**Table 1:** Chronology of main laws, regulations, and decrees regarding branded generics, generics, and general product quality and bioequivalence, 1994–2011

Year	Branded generics	Generics
	Law/regulation/content^a^	Law/regulation/content^a^
1994	Normative instruction n°1 - Requirements for the registration of similar medicines	
1996		Law 9279 - Regulates the rights and obligations regarding industrial property
1999		Law 9787 - Generics Law
		Resolution RDC 391 - States that for a product to be registered as generic there is a need to prove bioequivalence.
		Decree 3181 - Regulates Law 9787
2000	Resolution RDC 92 - Similar medicines can be marketed and identified by trade name or mark that distinguishes the products from those of other manufacturers	Resolution RE 74 - Approval of the first registrations of generic medicines
2001	Resolution RES 36 - Similar medicines obligated to start presenting commercial names; a deadline of 180 days was given to industries to comply	Resolution RDC 10 - Included a list of medicines that for safety reasons could not be registered as generic drugs; it is a revision of the RDC 391.
		Resolution RDC 47 - Regulating characteristics of the packaging of generics
		Decree 3961 Updates the definitions of similar, reference and generic drugs
2002	Resolution RDC 157 - Established the requirements for pharmaceutical equivalence studies for similar drugs	Resolution RDC 84 - Modifies the list of products identified in resolution RDC 10
2003	Resolution RDC 133 and RDC 134 - Regulations for the registration of similar medicines requiring products to undergo, by 2014, the same relative bioavailability and pharmaceutical equivalence testing, required from the beginning for generic medicines	Resolution RDC 135 - Approves the Technical Regulation for Generic Drugs; repeals RDC 391 and RDC 84
2007	Resolution RDC 17 - Similar medicines were obligated to present the same documents for licensing as needed for generics	Resolution RDC 16 - Approves the Technical Regulation for Generic Drugs; repeals RDC 135
2010	Resolution RES 16 - Amending and repealing legal texts related to the presentation prior to Anvisa protocol bioequivalence study; repeals RDC 17.	

^a^Laws, regulations and contents are available at: http://portal.anvisa.gov.br/legislacao

This paper aims to describe the changes in the private market for selected originator products, branded generics, and generics during the first 10 years of the Brazilian generics policy.

## Methods

We analyzed longitudinal data on sales of antibiotic, antidiabetic, and antihypertensive medicines by wholesalers to private retail pharmacies in Brazil collected by IQVIA® [18] between 1998 and 2010. The study design is a descriptive time series [19] with comparison series. We made descriptive comparisons of: 1) percentages of the market share, number of manufactures and sales volume; 2) brand median unit prices and medicine price ratios for branded generics and generics using the brand as reference price; 3) sales volumes of amoxicillin by types of manufacturers: all types, those who produce only branded generics, those who produce branded generics and generics and those who produce only generics and 4) number and percentage of manufacturers of generics and branded generics over time.

The data comprise a monthly audit of a universe of more than 58,000 private pharmacies. The sample design involves a stratified cluster sample of 11 regions [20, 21]. The sample size has changed somewhat over the study period. Since 2009, the sampling units have been 130 pharmacies (direct sales) and more than 400 wholesalers (indirect sales). The data include total quarterly sales volume and prices estimates for every product in the market, projected nationally based on the sample of pharmacies and wholesalers.

We analyzed the quarterly market share (proportions of the total volume of standard units sold) for originator, branded generics, and generic products from the second quarter (Q2) of 1998 through the first quarter (Q1) of 2010 within three widely used therapeutic classes (antibiotics, antidiabetics and antihypertensives). Generics data is available only after 2000, since they did not exist in 1998 and 1999, but the series of analyses started before (in 1998) to portrait the pharmaceutical market before the introduction of generics. The standard unit is the smallest unit of a product (ml, caps or cp). We also examined quarterly sales volume per capita (calculated by dividing sales volumes by annual population) and the number of manufacturers represented among the products analyzed.

The pharmaceutical classes selected were antidiabetics, antihypertensive and antibiotics. Hypertension and diabetes are chronic diseases that are prevalent in Brazil and are targeted by specific government programs to meet the demands of the population in terms of treatment. Antibiotics belong to a group of drugs of equal importance, usually of high cost, usually used in treatments for serious acute problems, which lead to the need for immediate treatment by the patient. The choice of these three groups serves as a model to demonstrate the evolution of the post-entry market of generics for important health problems to the Brazilian population and that generate great interest of the pharmaceutical industry.

Pharmacological groups are classified in the IQVIA® dataset using the EphMRA/PBIRG Anatomical Classification that is similar to the WHO/ATC Classification [22] (Table 2). We included in the analyses only the molecules included in therapeutic groups that had any generic products available in the Brazilian market during the study period and for which at least one generic manufacturer entered the market. Within these groups, we included all chemical entities in which there was at least one generic alternative on the market during the study period.

**Table 2:** Pharmacological groups and subgroups of medicines used in the study

Pharmacological Group	Pharmacological Sub-Group^a^
Anti-infectives for systemic use	subgroup J01/J1 – Antibacterials for systemic use/systemic antibacterials
Alimentary tract and metabolism medicines	subgroup A10 – Drugs used in diabetes
Cardiovascular system medicines	subgroups: C02/C2 – Antihypertensive (methyldopa); C03/C3 – Diuretics; C07/C7 – Beta blocking agents; C08/C8 – Calcium channel blockers /Calcium antagonists; C09/C9 – Agents acting on the renin-angiotensin system)

^a^ACT/EphMRA codes for pharmacological sub-groups included in the study

The IQVIA® classification of products includes the following types of licensing status: licensed brands, original brands, other brands, patent N/A, and unbranded. IQVIA® categorizes the relationship between companies and molecules. To identify the three groups of medicines of interest, we reclassified the data, maintaining the same classification for licensed brands, original brands, and other brand medicines, and analyzing, on a case by case basis, all medicines originally classified as patent N/A or unbranded. Our main objective was to identify which medicines from these two groups were generics, considering that all generics in Brazil are unbranded. We used three main sources of information for this reclassification: pack launch date provided in the IQVIA® dataset; the Brazilian agency of health regulation (ANVISA – ‘*Agência Nacional de Vigilância Sanitária*’) list which includes the date generics were approved to be commercialized in Brazil; and the Brazilian Website “Consulta remédios” [23] which includes all Brazilian medicines available in the market. We then classified products as: generics, originators and branded generics according to the following rules:

- Generics could only be drawn from the IQVIA® categories of Patent N/A or Unbranded (medicines with no brand names);
- All medicines with IQVIA® pack launch date before February/2000 maintained the original classification, because generics were launched in Brazil after this date;
- Medicines with missing IQVIA® pack launch date but within ANVISA list (date in which generics were approved to be commercialized in Brazil) were classified as generics;
- Medicines with missing date on IQVIA® and ANVISA dataset maintained their original IQVIA® classification;
- All medicines within the ANVISA list with an IQVIA® date after February/2000 were classified as generics;
- Medicines missing in the ANVISA list but with IQVIA® date after February/2000 were checked in the *Consulta Remédios* website. If the medicine was present, it was reclassified as generic, otherwise we kept the original IQVIA® classification.

For the price analysis, we compared the wholesaler prices of originator or licensed brand products with their generic and brand generic versions for the years 2002, 2006 and 2010. We used the unit price (US$) available for the first quarter of each year. We calculated the ratio (price of generic or branded generic divided by the reference price) of the unit prices for each molecule, for each manufacturer (generic and branded generic) that had a price available and we present the median value of the ratio in each period. The reference price was the original brand price; the licensed brand price was used when the brand was not available. The ratios are presented as percentages of the reference price. We only present the medicines for which we had price data for the three selected years and had at least price information for generics or branded generics for two of these years.

We selected oral formulations only for products that had oral formulations (whether or not they also had injectable formulations); if a molecule did not have an oral formulation, which happened for some antibiotics, we used only the injectable formulations.

Analyses were carried out using the statistical software Stata 10.0 and Microsoft Excel 2010. The use of the data was authorized by IQVIA®. The data are contained in a secondary database and do not involve human research subjects. The identities of the retail pharmacies and wholesalers in the sample were not part of the dataset.

## Results

We obtained data on 8559 products marketed in Brazil between 1998 and 2010. Of these, 2825 were systemic antibacterial (25.1% broad spectrum penicillin, 17.6% macrolide, 17.5% cephalosporin, 14.7% fluoroquinolone, 25.1% others), 448 medicines were used to treat diabetes (31.3% sulphonylurea, 25.5% biguanide, 25.9% insulin, 17.3% others) and 2113 to treat hypertension (5.3% methyldopa, 13.2% diuretic, 19.2% beta blocking agent, 16.7% calcium antagonist, 45.6% renin-angiotensin system agent).

**Fig. 1:**
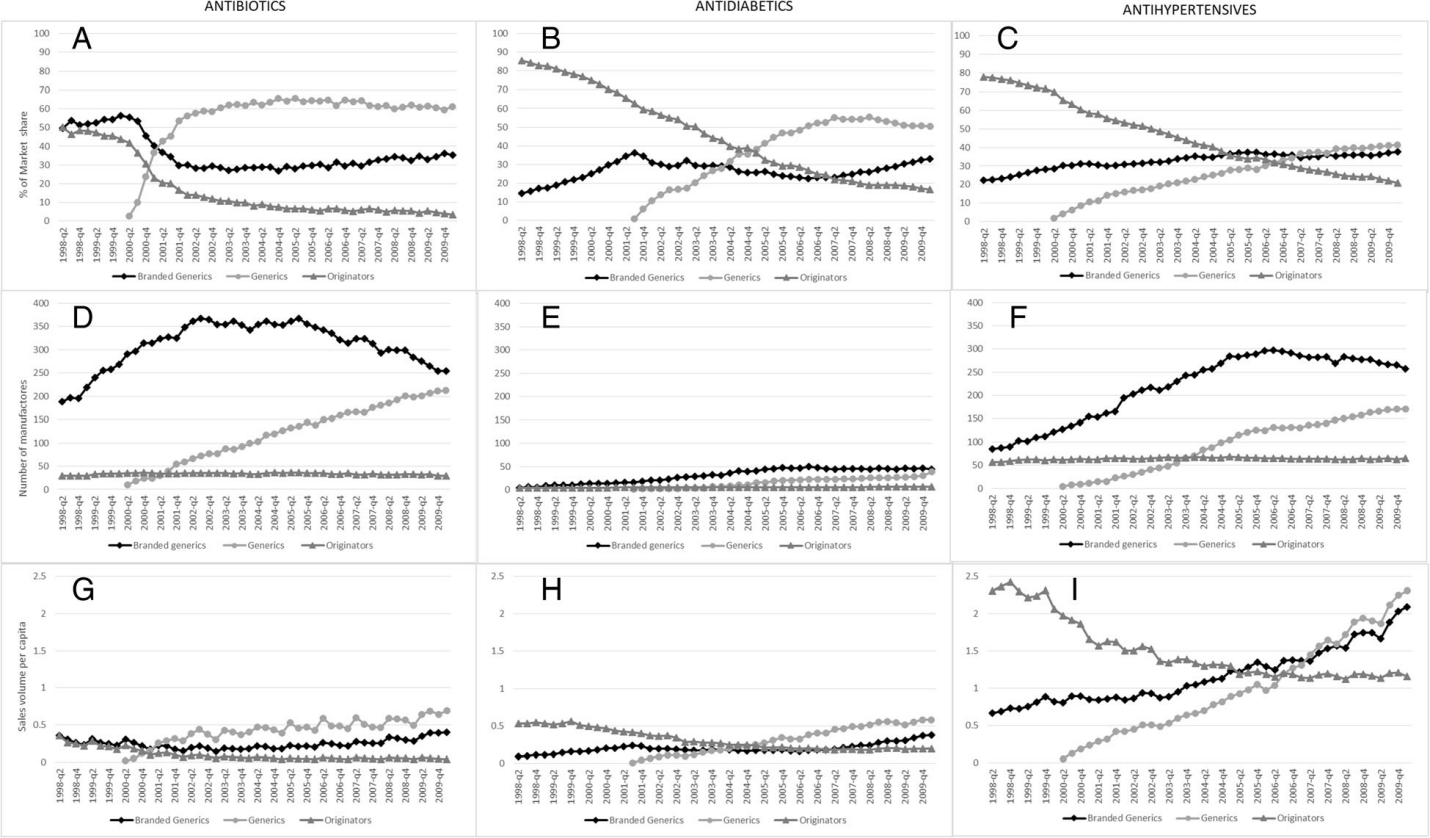
Quarterly proportion of market share (**a**-antibiotic; **d**-antidiabetic; **g**-antihypertensive) number of manufacturers (**b**-antibiotic; **e**-antidiabetic; **h**-antihypertensive), and sales volumes per capita (**c**-antibiotic; **f**-antidiabetic; **i**-antihypertensive) of antibiotics, antidiabetics and antihypertensive medicines in the ATC4 categories that have generic products in Brazil, 1998–2010

Longitudinal data on the market share, sales volume per capita, and number of manufacturers for the three pharmacological groups in the study are presented in Fig. 1. Results are described below for each group of medicines. Table 4 in Appendix shows trends in the numbers and percentages of manufacturers of generics, branded generics, or both over time.

For the three groups of medicines, the number of manufacturers of branded generics decreased from 2002 to 2010, while the number of manufacturers of generics increased. For antibiotics and antihypertensive, the number of manufacturers that produce both branded generics and generics has also increased over time. These trends were consistent in almost all subgroups of medicines included in the analysis.

### Systemic antibiotics

Almost all antibiotics for systemic use have generics in the market. We analyzed the seven groups with the largest sales volumes (almost 75% of the sample): oral and parenteral broad-spectrum penicillin; oral and parenteral fluoroquinolone; oral and parenteral cephalosporin, and macrolide.

Prior to the entry of generic products to the market, originators and branded generics had approximately equal market share (Fig. 1a). Market share changed very rapidly after the introduction of the first generic antibiotics in 2000-Q2. One year later, generics (43%) had overtaken both originators (20%) and branded generics (37%) in terms of market share. After initial declines, branded generic market share has remained relatively stable after 2001-Q4. The market share of originator antibiotics has declined consistently over time following introduction of generics, representing less than 5% of the market by the end of the study, compared to 60% for generics and 35% for branded generics. None of the manufacturers producing generic antibiotics were also manufacturers of originators.

There were consistently more manufacturers producing branded generics than generics or originators (Fig. 1b). However, after rising until 2002-Q3, the number of branded generics manufacturers remained stable until 2005-Q3 and declined afterwards. The number of manufacturers producing generic antibiotics, on the other hand, continued to rise during the whole period under study (Fig. 1b).

Generic antibiotic sales volume per capita has steadily increased from their introduction to the end of the study period, while the per capita sales volume of original brands has consistently decreased (Fig. 1c). After a decline following the launch of generics in the market, branded generics sales volume per capita has risen since 2004-Q2 but continued to remain below that of generics.

**Fig. 2:**
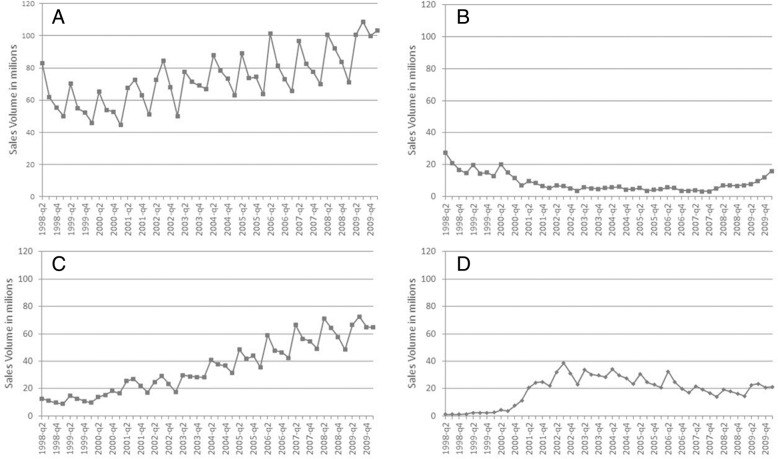
Sales volumes (IQVIA standard units) of amoxicillin by types of manufacturers: **a** all manufacturers; **b** manufacturers who produce only branded generics (*N* = 33); **c** manufacturers who produce branded generics and generics (*N* = 12) and **d** manufacturers who produce only generics (*N* = 8)

Figure 2 presents the amoxicillin case, as an example, which illustrates the general pattern of changes in the pharmaceutical market regarding the manufactures producing generics; similar trends are found for other medicines and therapeutic classes. Sales volume has increased overall (A) and among manufactures that produce branded generics and generics (C) but declined among those focusing their production on branded generics (B) or generics only (D).

As shown in Table 3, for approximately 50% of the antibiotic molecules analyzed, the branded generics and generics had lower prices than the originators. There was no consistent pattern of the percentage changes in prices. In 2010, some generics became more expensive than branded generics (Table 3).

**Table 3:** Brand or licensed brand median unit prices (US$) and medicine price ratios for branded generics and generics considering the brand or licensed brand as reference price, for antibiotics, antidiabetics and antihypertensives, in Brazil, 2002, 2006 and 2010

Selected medicines	2002			2006			2010		
	Originator	Branded generics	Generics	Originator	Branded generics	Generics	Originator	Branded generics	Generics
Antibiotics									
Penicillins									
Amoxicillin ^a, c^	1.02	72.0%	24.9%	1.48	54.9%	38.6%	1.53	46.9%	44.8%
Amoxicillin+Clavulanic acid ^a, c^	2.03	83.9%	66.8%	3.61	78.5%	49.1%	3.82	64.2%	75.4%
Ampicillin ^a, d^	1.06	75.2%	50.8%	1.49	49.4%	49.5%	1.82	35.6%	42.7%
Fluoroquinolones									
Ciprofloxacin ^a, c^	2.71	72.2%	**100.3%**	10.75	19.1%	31.2%	12.33	15.0%	19.6%
Levofloxacin ^a, d^	6.01	*	*	9.26	45.1%	54.4%	11.63	39.4%	43.1%
Norfloxacin ^a, d^	1.50	55.8%	59.4%	1.94	55.9%	60.0%	2.34	53.0%	57.3%
Cephalosporins									
Cefaclor ^a, d^	1.93	**148.8%**	*	2.39	**144.8%**	**107.6%**	3.57	*	72.2%
Cefadoxil ^a, c^	3.49	34.8%	43.6%	4.92	29.8%	36.9%	3.07	32.5%	67.1%
Cefalexin ^a, d^	1.53	78.2%	43.3%	1.74	81.7%	62.7%	2.13	64.0%	54.9%
Cefuroxime axetil ^b, c^	4.08	*	46.0%	4.99	*	42.5%	6.01	*	61.4%
Cefalotin ^b, d^	2.79	**106.7%**	**114.3%**	3.67	94.0%	80.7%	4.62	**101.3%**	65.7%
Cefazolin ^b, d^	5.58	**110.4%**	*	6.38	60.6%	*	4.29	**262.2%**	93.3%
Cefepime ^b, c^	19.10	*	*	25.05	**171.3%**	71.2%	57.79	87.8%	69.2%
Ceftazidime ^b, c^	29.97	56.6%	*	31.63	38.9%	47.2%	49.94	30.0%	30.0%
Ceftriazone ^b, c^	30.73	49.5%	25.4%	45.94	44.2%	23.5%	52.85	30.3%	30.0%
Macrolides									
Azitromycin ^a, d^	6.91	68.4%	99.3%	6.38	80.6%	**116.8%**	7.62	48.5%	83.1%
Claritromycin ^a, d^	3.12	**116.1%**	82.1%	3.43	88.6%	**118.2%**	4.11	64.0%	**119.3%**
Clindamycin ^a, c^	2.00	97.6%	60.8%	2.84	58.6%	53.9%	3.41	53.0%	52.8%
Erythromycin ^a, d^	0.64	**141.5%**	*	0.71	**139.8%**	*	0.81	85.8%	**111.7%**
Antidiabetics ^a, c^									
Glibenclamide	0.16	57.9%	66.9%	0.20	63.9%	63.7%	0.25	53.7%	63.5%
Glimepiride	0.38	**161.7%**	*	0.53	**118.1%**	**117.0%**	0.99	74.2%	78.8%
Antihypertensives ^a^									
Methyldopa ^c^	0.37	**167.3%**	**102.1%**	0.48	**123.7%**	78.4%	0.59	**108.5%**	72.3%
Diuretics									
Spironolacton ^c^	0.41	*	*	0.52	73.7%	64.4%	0.57	71.9%	65.7%
Furosemide ^c^	0.15	**106.8%**	86.0%	0.11	**218.5%**	**183.6%**	0.12	**174.6%**	**139.4%**
Chlorthalidone ^c^	0.19	88.3%	*	0.25	71.4%	66.0%	0.27	65.5%	79.7%
Amiloride+hydrochlorothiazide ^c^	0.20	97.1%	*	0.26	92.1%	68.1%	0.30	94.7%	64.7%
Betablockers									
Atenolol ^c^	0.44	68.9%	59.0%	0.61	53.3%	46.8%	0.70	46.9%	41.5%
Carvedilol ^c^	1.06	65.3%	*	1.57	38.1%	48.8%	1.91	32.7%	46.8%
Metoprolol ^c^	0.51	*	45.1%	0.36	*	89.2%	0.42	*	87.6%
Propranolol ^c^	0.11	63.0%	*	0.16	58.7%	56.8%	0.18	60.3%	57.1%
Sotalol ^c^	0.85	*	79.5%	1.33	65.4%	71.4%	1.58	75.8%	71.0%
Atenolol+Chlorthalidone ^c^	0.60	71.0%	*	0.83	58.7%	52.2%	0.94	55.3%	49.5%
Calcium antagonists									
Amlodipine ^c^	1.51	52.7%	50.5%	1.88	36.9%	41.6%	2.11	34.4%	46.2%
Diltiazem ^d^	0.45	**119.6%**	29.1%	0.55	**140.2%**	36.0%	1.01	**101.1%**	34.7%
Felodipine ^c^	1.20	*	*	1.62	76.5%	84.1%	1.92	*	50.1%
Verapamil ^c^	0.40	70.1%	91.4%	0.57	58.2%	52.1%	0.62	42.0%	56.8%
ACE inhibitors									
Captopril ^c^	0.58	54.0%	57.9%	0.87	38.5%	44.7%	1.09	30.6%	41.1%
Enalapril ^c^	0.97	39.4%	41.9%	0.46	97.9%	**113.8%**	0.33	**146.2%**	**170.5%**
Lisinopril ^c^	0.82	59.0%	61.6%	1.09	59.3%	62.3%	1.50	45.5%	53.7%
Ramipril ^c^	0.66	**122.6%**	*	2.66	40.7%	42.5%	3.23	30.9%	32.7%
Captopril+ hydrochlorothiazide ^c^	1.12	68.2%	*	1.57	73.1%	58.6%	1.79	46.4%	60.4%
Enalapril+ hydrochlorothiazide ^c^	1.04	65.3%	*	1.36	54.5%	61.0%	0.93	87.8%	**107.7%**
Lisinopril+ hydrochlorothiazide ^c^	1.10	**131.4%**	*	1.40	60.9%	**108.2%**	1.60	69.0%	*
Ramirpil+ hydrochlorothiazide ^c^	2.18	48.1%	*	3.08	46.6%	*	3.49	37.4%	44.8%
Losartan ^d^	1.76	62.9%	*	2.48	40.2%	36.4%	1.06	68.4%	63.5%
Losartan+ hydrochlorothiazide ^d^	1.76	75.2%	*	2.49	35.6%	55.1%	1.54	61.8%	82.1%

The time points are the first quarter of the respective year

*no unit price for generic or branded generic available

^a^Oral presentations

^b^Injectable preparations

^c^Original brand

^d^Licensed brand

Medicine price ratios for branded generics and generics considering the brand or licensed brand as reference price with percentages higher than 100% were highlighted in boldface

For the cephalosporins, in general, branded generics had higher prices than originators. In the 2010, cefazolin branded generic had a price more than 2 times higher (262%) than the originator (Table 3).

Finally, branded generic prices have fallen substantially over time for all macrolides. The price of generics increased over time, eventually exceeding the price of the originator medicines for clarithromycin (2006 and 2010) and azithromycin (2006) (Table 3).

### Medicines used in diabetes

Antidiabetics with generics were sulphonylureas and biguanides which represented 56.7% of all antidiabetic medicines.

Prior to the appearance of generics, originators dominated the market, corresponding to around 85% of sales volume (Fig. 1d). From 1998 to the 2001-Q3, there were concomitant rises in the market share of branded generics and declines in the sales of originators. After the introduction of generics in the market, the market share of originators continued to decline, while branded generics no longer increased as generics began to increase. However, the increase in generic antidiabetic market share was more gradual than for antibiotics, and it did not surpass 50% until 2007. By 2010, generics corresponded to half of all sales, branded generics to just over 30%, and originators had fallen to below 20%.

There was a rapid rise in the number of manufacturers producing branded generics in the antidiabetic class, and this number stabilized from 2005-Q3 onwards (Fig. 1e). Following the introduction of generic antidiabetic products in the market in 2001-Q3, there was a consistent increase in the number of manufacturers producing them. Among the manufacturers of the three molecules included in the study, one also started to produce a generic equivalent in 2010-Q1. Few manufacturers of antidiabetics produced generics and branded generics simultaneously.

Generic sales volumes per capita have increased steadily since mid-2001 when the first generics entered the market, while there has been a gradual decline in the sales volumes of originators that began even prior to the introduction of generics (Fig. 1f). The sales volumes of branded generics declined slightly from 2001 to 2006, but sales have risen steadily since that period.

The prices of glimepiride branded generics experienced important reductions over time (Table 3), especially after the introduction of generics. Glibenclamide, on the other hand, experienced only small fluctuations in prices, both for generics and branded generics. For both antidiabetic medicines, the prices of branded generics and generics were quite similar during the period analyzed.

### Medicines used in hypertension

The market share for both branded generics and generics to treat hypertension tended to increase until 2006-Q1 (Fig. 1g). After that, market share of generics continued to increase, reaching a high of 40%, whereas branded generics market share stabilized at around 35%. Originators declined consistently, reaching a low of 23% market share.

In 2000-Q2, the first antihypertensive generics were launched in the market, and the number of manufacturers producing generics increased steadily over time. Nevertheless, the number of manufacturers producing branded generic has remained consistently higher than generics. From 2006-Q2 onwards, there was a decline in the number of manufacturers producing branded generics (Fig. 1h). Among the five ATC2 groups of medicines used to treat hypertension in the study, we identified 30 molecules with generics. Only two manufacturers producing originators also produced generics (furosemide and ramipril, both from the same manufacturer). There were 21 molecules for which the same manufacturer produced generics and branded generics.

Antihypertensive originator sales volumes per capita decreased rapidly during the entire period, while per capita sales volumes of branded generics and generics increased. After 2007-Q2, sales volumes of generics exceeded those of branded generics (Fig. 1i).

Price trends over the years for the subclasses had very different patterns (Table 3). For instance, methyldopa branded generic prices have always been higher than those of the originator. Generics started with a price similar to originators but experienced a reduction overtime.

In the diuretics, except for furosemide, the originator prices have risen. For furosemide, branded generic versions have always had a higher price than the originator. Even though the price ratio for generics is not as high as it is for the branded generics, it was still higher than the originator price.

Betablockers did not experience meaningful fluctuations in prices during the period analyzed, except for metoprolol. For this medicine, which had no branded generics available in the Brazilian market, the generics price increased in 2006 to 89.2% of the originator’s price and remained mostly the same in 2010.

The calcium antagonists subclass exhibited two different patterns of price variation. For amlodipine and verapamil as examples of the first pattern, branded generics and generics had price reductions while originators had price increases from 2002 to 2010. For diltiazem as an example of the second pattern, branded generics were the most expensive version, even with increases in the originator’s price, and prices were about equal in 2010.

Finally, the ACE inhibitors experienced interesting patterns of price variations from 2002 to 2010. Overall, originator prices increased from 2002 to 2010, except for enalapril; in contrast, enalapril + hydrochlorothiazide, losartan and losartan + hydrochlorothiazide had lower originator prices in 2010.

## Discussion

The introduction of generics in the Brazilian market led to large decreases in the sales volumes of originators in the three therapeutic classes examined. Changes in market share started as soon as the first generics in each class were introduced into the market. After 10 years of the generics policy, generics represented the largest market share in all classes, although the proportion varied across pharmacological groups and molecules. The speed of the transition to generics has varied by class, with the shift occurring most rapidly for antibiotics, followed by antidiabetics and antihypertensives. The introduction of generics was much more impactful for antibiotics than for the other groups. It is possible that the growth of generics is related to whether the drugs are used to treat chronic versus acute illnesses. For a country unused to generics, it may be more difficult to change use of established products for chronic therapy. Repeated prescriptions and purchases without a prescription will tend to be of the same rather than another product with a different name [24–26].

Several manufacturers that originally produced branded generics have started to produce generics of the same molecules, accounting for 25, 12 and 20% of the manufacturers of antibiotics, antidiabetics and antihypertensives, respectively. This is likely an attempt to retain or increase market share [27]. A good example is amoxicillin where sales volumes increased overall and for manufacturers producing both branded generics and generics but declined for manufacturers producing branded generics only. Furthermore, the sales volumes of manufacturers producing generics only has decreased over time.

We expected that the number of branded generics in the market would continue to decline over time; however, despite increases in generic market share over the years, by 2011 branded generics continued to be the most widely sold in Brazil [28].

The generic law occurred in Brazil in a historical moment where counterfeit medicines were increasing [29]. Generic medicines brought the possibility of having more affordable medicines with the same quality that the original. Prior to the approval of the generic law, local pharmaceutical companies could copy originator medicines without presenting any therapeutic equivalence test [30]. With the introduction of generics, there was an increase in the inspection of good manufacturing practices [29, 30]. Thus, the relevance of our finding, that even with increases costs due to the need of bioequivalence tests to register the generics, the pharmaceutical market turned in this direction, with the reduction of branded generics. Diverse interests of public and private sector stakeholders might shaped generic drug policy and its implementation [30]. Considering the international context, in Brazil we presented similar trends as others low and middle-income countries (LMICs). The market share of originators substances had the largest decrease over time when compared to the market share of their counterpart generic versions [28].

By law, it is mandatory that generic medicines enter the market at least 35% below the price of the originator [31]. This regulation is not applicable to branded generics. However, our results do not always show this relationship. Instead, we observe cases in which generics were more expensive than originators. This is likely because the price regulation in Brazil applies to ceiling prices, the maximum prices allowed for each medicine to be sold by wholesalers to pharmacies and by pharmacies to consumers. These ceiling prices are established at the time of registration. Our data reflect selling prices, which are lower than these ceiling prices [17, 32]. Furthermore, there is no legal mechanism to reduce these ceiling prices over time [32].

When generic prices are higher than originator prices [17, 33], wholesalers may be applying discounts to originators and branded generics to retain market share in the face of lower cost generics. With the increasing acceptance of generics by prescribers and consumers, it is possible that generics become more expensive due to a public image of having better quality than branded generics.

In Brazil, the prevalence of generic medicines use was 45.5% in 2014. In the private sector, a relevant part of the population is choosing generic medicines, due to its availability as an option for the most medicines used by the population, which is an evidence that the generic law in Brazil has increase access to medicines [34].

Our study has several limitations. It is possible that some misclassification took place in the process of reclassifying products from the IQVIA® categories. Reasons may include: missing ANVISA date or IQVIA® launch date, ANVISA date that occurs after the IQVIA® launch date, or ambiguity in product identity due to mergers of manufacturers. Some products may also have been misclassified in the IQVIA® dataset. To minimize misclassification, we used consistent rules for classification. Another limitation is that IQVIA® data represent only a sample of suppliers and facilities; however, this information is submitted to a validation process to assure the accuracy of the national estimates. An additional limitation is that IQVIA® collects price data from wholesalers, which may not reflect commercial deals further along the supply chain, such as discounts to the pharmacy. These discounts mean that the originator or branded generics can be sold at prices more similar to that of the generics. The sales data may not only reflect the effect of policy per se but also the effect of commercial reactions to that policy.

## Conclusion

In conclusion, entry of generic products following the Brazilian Generic Medicines Law resulted in important reductions in market share for originator products, price reductions for branded generics and originators, and increases on the total number of manufactures in the therapeutic classes selected. Speed of market appearance and uptake of generics varied by pharmacological group. The 1999 Generics Law changed the dynamics of the pharmaceutical market during its first 10 years of implementation. Future studies should examine continuing changes in the market as consumers gain greater confidence in generic products and manufacturers develop new marketing strategies.

## Appendix

**Table 4:** Number and percentage^c^ of manufacturers of generics and branded generics by licensure type over time^b^

Selected medicines^a^	2002						2006						2010					
	Branded generics		Generics		Generics + Branded generics		Branded generics		Generics		Generics + Branded generics		Branded generics		Generics		Generics + Branded generics	
	N	%	N	%	N	%	N	%	N	%	N	%	N	%	N	%	N	%
Antibiotics																		
Penicillins	36	78.3	3	6.5	7	15.2	32	68.1	6	12.8	9	19.1	15	41.7	7	19.4	14	38.9
Fluoroquinolones	29	85.3	2	5.9	3	8.8	36	69.2	9	17.3	7	13.5	22	47.8	12	26.1	12	26.1
Cephalosporins	19	73.1	2	7.7	5	19.2	20	57.1	8	22.9	7	20	11	39.3	7	25	10	35.7
Macrolides	29	82.9	1	2.9	5	14.3	31	72.1	2	4.7	10	23.3	23	57.5	7	17.5	10	25
Antidiabetics																		
Glibenclamide	11	91.7	1	8.3	0	0	15	71.4	5	23.8	1	4.8	12	57.1	8	38.1	1	4.8
Glimepiride	2	100	0	0	0	0	9	69.2	3	23.1	1	7.7	6	35.3	9	52.9	2	11.8
Metformin	4	66.7	1	16.7	1	16.7	13	56.5	9	39.1	1	4.3	13	41.9	15	48.4	3	9.7
Antihypertensives																		
Methyldopa	12	92.3	1	7.7	0	0	13	76.5	2	11.8	2	11.8	7	63.6	2	18.2	2	18.2
Diuretics	20	90.9	1	4.5	1	4.5	27	77.1	5	14.3	3	8.6	22	68.8	6	18.8	4	12.5
Betablockers	13	76.5	2	11.8	2	11.8	17	60.7	6	21.4	5	17.9	15	48.4	9	29	7	22.6
Calcium antagonists	18	81.8	2	9.1	2	9.1	24	63.2	8	21.1	6	15.8	16	48.5	11	33.3	6	18.2
ACE inhibitors^d^	26	78.8	3	9.1	4	12.1	37	71.2	8	15.4	7	13.5	33	62.3	12	22.6	8	15.1

^a^Included are only antidiabetic, antihypertensive and antidiabetic molecules which had generics in the market during the study period

^b^Time points are the first quarter of the respective year

^c^Denominators are the total number of manufacturers of branded generics and generics in each period

^d^*ACE* = Angiotensin-converting enzyme
